# Unravelling the Homology between Calycine Glands in Malpighiales: New Data from Basal Malpighiaceae

**DOI:** 10.3390/plants13121654

**Published:** 2024-06-14

**Authors:** Stéphani Karoline Vasconcelos Bonifácio, André Márcio Araújo Amorim, Élder Antônio Sousa Paiva, Denise Maria Trombert Oliveira

**Affiliations:** 1 Departamento de Botânica, Instituto de Ciências Biológicas, Universidade Federal de Minas Gerais, Belo Horizonte 31270-901, MG, Brazil; stephanivasconcelos@gmail.com (S.K.V.B.); epaiva@icb.ufmg.br (É.A.S.P.); 2 Departamento de Ciências Biológicas, Universidade Estadual de Santa Cruz, Ilhéus 45600-970, BA, Brazil; amorim.uesc@gmail.com

**Keywords:** calycine glands, elaiophore, extrafloral nectary, floral glands, floral rewards

## Abstract

Discussing homology relationships among secretory structures remains a relatively underexplored area in botanical research. These structures are widely dispersed within Malpighiales, one of the largest orders of eudicots. Within Malpighiales, both extranuptial and nuptial nectaries are present, and they do not seem homoplastic or share evolutionary connections. Particularly in Malpighiaceae, extensive research has focused on the ecological interactions mediated by glands. Botanists largely agree that elaiophores in sepals of Neotropical Malpighiaceae have evolved from extrafloral nectaries on leaves. However, the evolutionary origin of elaiophores has yet to be thoroughly examined, particularly in comparison to outgroups. This study provides empirical evidence on the ontogeny of elaiophores and investigates their evolutionary origins and homology relationships across different lineages of Malpighiales using comparative anatomy. Our findings suggest that elaiophores are likely homologous to extranuptial nectaries found in sepals of other Malpighiales lineages, originating from nectaries on leaves. This discussion is a starting point for future studies exploring the evolution of nectaries found in flowers, whether extranuptial or nuptial, and their potential origins from nectaries in vegetative organs such as leaves. Understanding these relationships could shed light on the selective pressures influencing floral morphologies.

## 1. Introduction

Identifying homology relationships among secretory structures poses a challenging endeavour. However, understanding the evolution of these structures in taxonomic groups can provide valuable insights into phylogenetic relationships [1]. In Malpighiales, for example, secretory structures are widely distributed among clades [1,2,3,4,5,6,7] whose phylogenetic relationships remain unresolved [8]. There is a record of a great diversity of glands within the Malpighiales, encompassing various types, including nectaries, colleters, resin glands [9], osmophores [7], laticifers [10], and elaiophores [11,12]. Elaiophores, in particular, are glands responsible for oil secretion and are notably situated on the abaxial surface of sepals in Neotropical species of Malpighiaceae [13,14,15]. The significance of elaiophores in the evolution of Malpighiaceae is widely recognised and attributed to their role in maintaining mutualistic relationships with oil-collecting bees [13,14,16]. Notably, variation exists in the number of calycine glands across species, with some featuring up to ten elaiophores (two per sepal). However, it is important to highlight the presence of species with an eglandular calyx and those exhibiting both glandular and eglandular morphotypes [13,14,16].

Malpighiaceae is a tropical family with about 1300 species and 78 genera of trees, shrubs, and lianas in the Old and New Worlds [17,18,19,20]. In contrast to the morphological diversity of their fruits, Neotropical species are characterised by their homogeneous floral morphology, which encompasses mostly yellow, zygomorphic, pentamerous flowers, with a pair of oil glands (elaiophores) at the base between two adjacent sepals [13,14,16]. The Malpighiaceae family is considered monophyletic, but synapomorphies are unknown for many of its lineages, which are treated informally as clades [17]. The galphimioid and byrsonimoid clades occupy the first branches that diverged in Malpighiaceae [18] and retained characters presumably plesiomorphic for the family, such as the presence of a calyx with ten elaiophores. Because of their phylogenetic position, species from both clades are relevant to broaden our understanding of the evolution of the family.

The primary challenge in examining homology relationships among secretory structures lies in the need for standardised terminology and clear conceptual boundaries within the literature, particularly concerning nectaries. Different criteria exist for classifying nectaries, with some emphasising topography, others prioritising functional aspects, and some combining both criteria. This lack of uniformity complicates comparative analyses and hinders precise interpretation. The term extrafloral nectary clearly encompasses the redundant concept that they are structures outside the flower. The classification of nectaries proposed by Caspary [21] was primarily based on the position of the structure within the plant body. It should not raise doubts: nectaries in floral parts are called floral nectaries, while those in other plant parts are called extrafloral nectaries. However, this topographic classification sensu Caspary [21] may not always suffice, as the varied nectaries within flowers can serve different functions, with some aiding in pollinator attraction and others not. An alternative classification proposed by Federico Delpino [22] focuses on functional aspects. According to Delpino, nectaries that mediate pollination, especially attracting pollinators, are termed “nuptial nectaries”, whereas those not involved in this function are termed “extranuptial nectaries”. While Delpino’s functional classification offers insights into the diverse roles of nectaries, its practical application poses challenges, requiring extensive field observations and reproductive biology studies to ascertain pollinator relationships with the nectaries present in the flower. 

To simplify the adoption of Delpino’s framework, some authors adopt a hybrid approach, using the term “floral” for nectaries involved in pollination and “extrafloral” for those not involved, aligning with Delpino’s definitions of nuptial and extranuptial nectaries while retaining Caspary’s terminology. However, this hybrid approach has led to terminological confusion and misunderstandings. To mitigate such issues and ensure clarity, we chose to preferably use here the classification of nectaries proposed by Delpino (nuptial and extranuptial), avoiding the ambiguous use of “extrafloral” to refer to extranuptial nectaries within floral structures.

In Malpighiaceae, elaiophores are considered homologous to extrafloral nectaries (sensu Caspary [21]), which are extensively distributed within the family and exclusively found in vegetative organs [14,15,23,24,25]. These glands are considered homologous to each other due to their widespread occurrence among species and their shared structural features. In both cases, a palisaded epidermis overlaid by a cuticle layer that encloses the exudates delimits the glands [15]. It is hypothesised that during the evolutionary history of Malpighiaceae, there was a transition in the exudate type secreted by the calycine glands. In Neotropical species, this transition shifted from nectar to oil [14,15]. Conversely, this pattern is reversed in Paleotropical species, considered more recent than their Neotropical counterparts, with the presence of calycine nectaries (extranuptial) [25]. 

Vogel’s proposal suggesting homology between nectaries found in vegetative organs and calycine elaiophores [15] predates phylogenetic studies that have since clarified the relationships within Malpighiaceae in the order Malpighiales [8,26]. Furthermore, the recognised kinship relationships have evolved, differing from those previously established. Consequently, the phylogenetic origin of Malpighiaceae elaiophores has yet to be comprehensively addressed within the context of the current understanding of Malpighiales.

Vasculature corresponds to the more conservative floral trait, often adopted to explore ancestral questions [27,28]. Given that basal clades are anticipated to possess characters more akin to the common ancestor among families, analysing the floral vascular anatomy of lineages that initially diverged into Malpighiaceae can shed light on the emergence of these structures. Thus, this study aims to elucidate the evolutionary origins of elaiophores by examining the vasculature and ontogeny of these glands within two basal clades of Malpighiaceae, namely, byrsonimoid and galphimioid, and comparing them to other families within Malpighiales.

## 2. Results

### 2.1. Elaiophore Vasculature in the Galphimioid and Byrsonimoid Clades

Species of the galphimioid and byrsonimoid clades exhibit three distinct patterns of vasculature where elaiophores are vascularised by lateral sepal traces. These lateral traces, described in detail in this study, originate from either (1) lateral sepal–petal complexes, (2) sepal–petal–antipetalous stamen complexes, or (3) median sepal–petal complexes.

**Figure 1:**
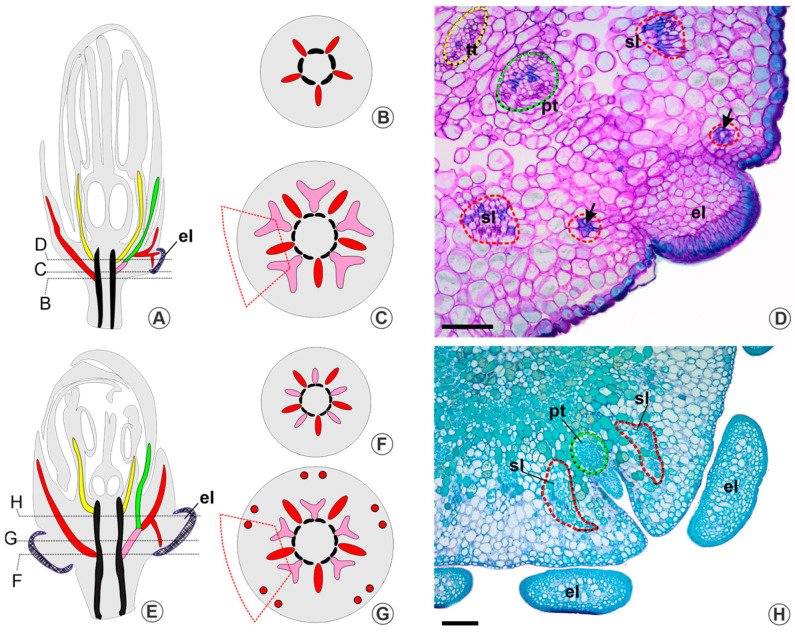
Calycine vasculature in *Galphimia australis* (**A**–**D**) and *Verrucularia glaucophylla* (**E**–**H**). The hatched areas represent elaiophores, while colours represent vascular cylinder (black), sepal–petal complexes (pink), sepal traces (red), petal traces (green), and stamen traces (yellow). (**A**) Diagram of median longitudinal reconstruction of the floral bud showing the vasculature of the elaiophores; note that the elaiophores only extend in the region opposite the petal (black dashed lines point to the approximate position of the cross-sections indicated by capital letters). (**B**,**C**) Diagrams of the receptacle showing the emission of the median sepal traces and sepal–petal complexes (the trapezium indicates the vasculature emitted to one sepal). (**D**) Photomicrograph of a floral bud in cross-section showing the elaiophore between two adjacent sepals in the region above the emission of the lateral sepal traces (arrow: basipetal trace). (**E**) Diagram of median longitudinal reconstruction of the floral bud showing the vasculature of the elaiophores (black dashed lines point to the approximate position of the cross-section). (**F**,**G**) Diagrams of the receptacle showing the emission of the median sepal traces and sepal–petal complexes (the trapezium indicates the vasculature emitted to one sepal; red circles represent basipetal traces with xylem outer rather than phloem). (**H**) Photomicrograph of a cross-section of the floral bud showing two elaiophores in the margins of two adjacent sepals in the region above the emission of the lateral sepal traces. Abbreviations: el, elaiophore; gl, calycine glands; pt, petal trace; sl, lateral sepal traces; tt, antipetalous stamen trace. Scale bars = 200 μm.

In the first pattern of vasculature, in species in which there are sepal–petal complexes (genera *Galphimia*, *Verrucularia*, *Byrsonima*, and *Blepharandra*), the distribution of lateral sepal traces varies according to the number and size of calycine glands occurring in the species. For instance, in *Galphimia*, we examined *G. australis*, which exhibits two morphs (glandular and eglandular), and another completely eglandular species (*G. brasiliensis*). In both species, the median sepal traces are emitted first (Figure 1A,B), followed by the sepal–petal complexes between them. These vascular complexes branch laterally, originating two lateral sepal traces and one petal trace. The lateral traces then migrate to the periphery of the receptacle towards two adjacent sepals (Figure 1C). If glands are present, the lateral traces irrigate them. These glands are elaiophores and constitute small protrusions in the receptacular region, covered by a palisade epidermis (Figure 1D). In both species, the total number of glands per flower varies (with a maximum of four recorded in our sample), never located in pairs in the margin of adjacent sepals. In *G. australis*, the two lateral sepal traces adjacent to the elaiophore irrigate it basipetally (Figure 1D). This vasculature pattern persists even when glands are absent, as observed in the eglandular morph. In *G. brasiliensis*, an eglandular species, the pattern of traces for sepals and petals remains identical, but basipetal traces are not formed. For a detailed illustration of this vasculature pattern, see Supplementary Material (Figure S1A–H).

*Blepharandra hypoleuca*, *Byrsonima stipulacea*, *Byrsonima triopterifolia*, and *Verrucularia glaucophylla*, in turn, exhibit a 10-glandular calyx, with two elaiophores per sepal, which can be fused at the base. An eglandular morph of *Byrsonima stipulacea* maintains the vasculature pattern observed in the glandular morph. Each sepal–petal complex branches laterally, emitting two lateral sepal traces, and each of these emits a basipetal trace, which irrigates the corresponding elaiophore (Figure 1E–H). For a detailed illustration of this vasculature pattern, see Supplementary Material (Figures S1I–N, S2 and S3).

**Figure 2:**
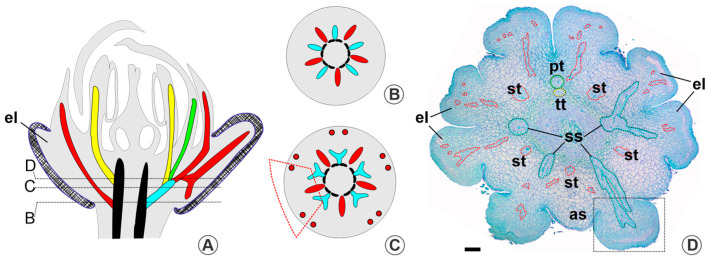
Calycine vasculature of *Spachea elegans*. The hatched areas represent elaiophores, while colours represent vascular cylinder (black), sepal–petal–antipetalous stamen complexes (light blue), sepal traces (red), petal traces (green), and stamen traces (yellow). (**A**) Diagram of median longitudinal reconstruction of the floral bud showing the vasculature of the elaiophores (black dashed lines point to the approximate position of the cross-section). (**B**,**C**) Diagram of the receptacle showing the emission of the median sepal traces and sepal–petal–antipetalous stamen complexes (the trapezium indicates the vasculature emitted to one sepal; red circles represent basipetal traces with xylem outer rather than phloem). (**D**) Photomicrograph of a floral bud in cross-section showing elaiophores at the position of the complexes branching; note that one of the elaiophores adjacent to the anterior sepal receives two lateral sepal traces from the sepal–petal–antipetalous stamen complex. Abbreviations: as, anterior sepal; el, elaiophore; pt, petal trace; ss, sepal–petal–antipetalous stamen complex; st, median sepal trace; tt, antipetalous stamen trace. Scale bars = 200 μm.

In the second vasculature pattern, observed in *Lophanthera lactescens* and *Spachea elegans*, the elaiophores are also supplied by lateral sepal traces originating from sepal–petal–antipetalous stamen complexes (Figure 2A). In these two species, the flower has eight elaiophores: four sepals have two glands, and the anterior sepal is eglandular. At the base of the receptacle, five median sepal traces and five sepal–petal–antipetalous stamen complexes migrate simultaneously from the vascular cylinder (Figure 2B). Each median sepal trace forms the median sepal bundle, while each sepal–petal–antipetalous stamen complex divides and irrigates an antipetalous stamen, a petal, and laterally, two sepals; each of the two sepals receives a trace that irrigates the gland (Figure 2C,D). In these eight-glandular species, where the anterior sepal lacks glands, the lateral traces of this sepal supply the elaiophores of the adjacent sepals (Figure 2D). In this way, the latero-posterior sepals receive three traces, the latero-anterior sepals receive four traces (the three of them plus a trace that would go to the anterior sepal), and the anterior sepal receives a single trace. The petals and stamens receive a single vascular trace each. For a detailed illustration of this vasculature pattern, see Supplementary Material (Figure S4). 

**Figure 3:**
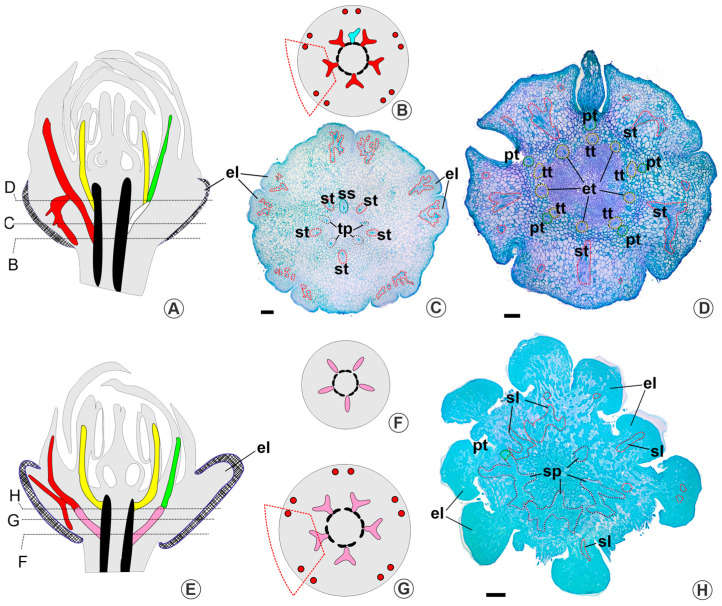
Calycine vasculature of *Lophanthera longifolia* (**A**–**D**) and *Diacidia aracaensis* (**E**–**H**). The hatched areas represent elaiophores, while colours represent vascular cylinder (black), sepal–petal–antipetalous stamen complex (light blue), petal–antipetalous stamen complexes (white), sepal traces (red), petal traces (green), and stamen traces (yellow). (**A**) Diagram of median longitudinal reconstruction of the floral bud showing the vasculature of the elaiophores (black dashed lines point to the approximate position of the cross-section). (**B**) Diagram of the receptacle showing the emission of the median sepal traces and sepal–petal–antipetalous stamen complex; note that there is one vascular complex that emits only a lateral sepal trace, while the lateral sepal traces come from the median sepal traces in the other sepals (the trapezium indicates the vasculature emitted to one sepal; red circles represent basipetal traces with xylem outer rather than phloem). (**C**) Photomicrograph of a floral bud in cross-section in the distal region of the receptacle; note that the elaiophores are irrigated by the peripheral vasculature of the receptacle. (**D**) Photomicrograph of a floral bud in cross-section showing the branching of the median sepal traces. (**E**) Diagram of median longitudinal reconstruction of the floral bud showing the vasculature of the elaiophores (black dashed lines point to the approximate position of the cross-section). (**F**,**G**) Diagrams of the receptacle showing the emission of the median sepal traces modified in sepal–petal complexes; note that no traces are emitted in the regions between sepals (the trapezium indicates the vasculature emitted to one sepal; red circles represent basipetal traces with xylem outer rather than phloem). (**H**) Photomicrograph of a floral bud in cross-section showing the branching of the sepal–petal complexes; note the separation of a petal trace from the complex. Abbreviations: el, elaiophore; et, antisepalous stamen; pt, petal trace; sl, lateral sepal traces; sp, median sepal–petal complex; ss, sepal–petal–antipetalous stamen complex; st median sepal trace; tp, petal–antipetalous stamen complex; tt, antipetalous stamen trace. Scale bar = 200 μm.

The third vasculature pattern is evident in *Diacidia aracaensis* and *Lophanthera longifolia*, both with ten elaiophores. In *L. longifolia*, elaiophores receive vasculature through lateral sepal traces originating from median sepal traces (Figure 3A), except for the elaiophore adjacent to the posterior petal, which is supplied by a sepal–petal–antipetalous stamen complex (Figure 3B). In the receptacle, five median sepal traces are emitted. Even before their branching, peripheral vasculature supplying the elaiophores can be seen (Figure 3B). From the sepal–petal–antipetalous stamen complex, a single lateral sepal trace, the posterior petal trace, and an antipetalous stamen trace are emitted (Figure 3C,D). For a detailed illustration of this vasculature pattern, see Supplementary Material (Figure S5). 

In *Diacidia aracaensis*, the ten elaiophores receive vasculature from five median sepal-petal complexes (Figure 3E). These complexes emerge directly from the base of the receptacle (Figure 3F), branching laterally to originate the two lateral traces of the sepal and a petal trace (Figure 3G). Subsequently, the lateral traces migrate towards the periphery of the receptacle, reaching the two sepal glands (Figure 3H). For a detailed illustration of this vasculature pattern, see Supplementary Material (Figure S6).

### 2.2. Ontogeny of Elaiophores in the Galphimioid and Byrsonimoid Clades

**Figure 4:**
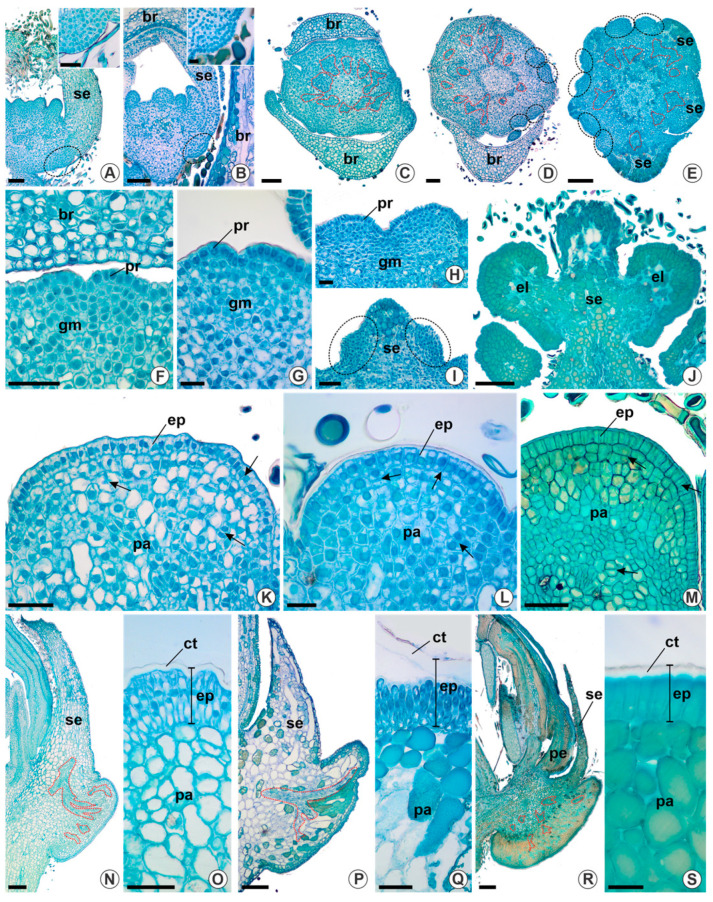
Elaiophores ontogeny in *Lophanthera lactescens* (**A**,**C**,**F**,**K**,**N,O**), *Verrucularia glaucophylla* (**B**,**D**,**G**,**L**,**P**,**Q**), and *Byrsonima triopterifolia* (**E**,**H**–**J**,**M**,**R**,**S**). Longitudinal (**A**,**B**,**N**,**P**,**R**) and transversal (**C**–**M**,**O**,**Q**,**S**) sections. Red dashed lines indicate procambial tissue and black ellipses indicate glandular primordia. (**A**,**B**) Young floral buds with glandular primordia; note the dense protoplast and prominent nuclei in the highlights at the top of the figures. (**C**–**I**) Ontogenetic early stages of calycine glands during floral development; note the marginal position of the glands in (**I**). (**J**–**M**) Expansion stage of the calycine glands (arrows: cell divisions). (**N**–**S**) Final stage of elaiophore maturing; observe the cuticle covering epidermis in palisade. Abbreviations: br, bracteole; ct, cuticle; ep, epidermis; el, elaiophore; gm, ground meristem; pa, subglandular parenchyma; pe, petal; pr, protodermis; se, sepal. Scale bars = 20 μm (**E**–**G**, **J**,**Q**,**S**), 50 μm (**D**,**I**,**K**,**M**,**O**), 100 μm (**A**–**C**,**H**), and 200 μm (**L**,**N**,**P**,**R**).

The ontogeny of elaiophores in *Verrucularia glaucophylla*, *Lophanthera lactescens*, and *Byrsonima triopterifolia* is similar. The formation of these glands begins after the expansion of the sepals and petals. Precursor cells for elaiophores are observed in the proximal region of sepals, adjacent to their attachment to the receptacle, distinguished by their dense protoplast and prominent nuclei (Figure 4A,B). From this stage onwards, glands are recognised as two protrusions at the margin of sepals, with discernible procambial strands (Figure 4C–E) even in floral buds. 

The growth of elaiophores is marked by cell divisions in the ground meristem and protodermis (Figure 4F–H), accompanied by the elongation of the procambial strands. In *B. triopterifolia*, the glands are restricted to the margins of the sepals (Figure 4I) and as cell divisions occur in different planes, the glands project from the margins of the sepals towards the proximal region of the receptacle (Figure 4J). Subsequently, across all species, the next stage is marked by the growth of glands, a phase characterised by cell divisions and expansion in different planes, particularly in the layers immediately beneath the epidermis. This phase coincides with the increase in the volume of the glands through cell divisions (Figure 4K–M), especially in the anticlinal plan. Ultimately, the glands consist mainly of a conspicuous epidermis and a broad parenchyma in which numerous phenolic idioblasts are observed (Figure 4M). Each gland has a unistratified epidermis, formed by anticlinally elongated cells and covered by a conspicuous cuticle (Figure 4N–S); the absence of stomata stands out. 

### 2.3. Homology between Elaiophores, Floral, and Extrafloral Nectaries in Malpighiales

A summary of the data obtained from the literature for calycine glands in Malpighiales families, except Malpighiaceae, is provided in Table 1. Information on Malpighiaceae basal groups is provided in this article. 

**Figure 5:**
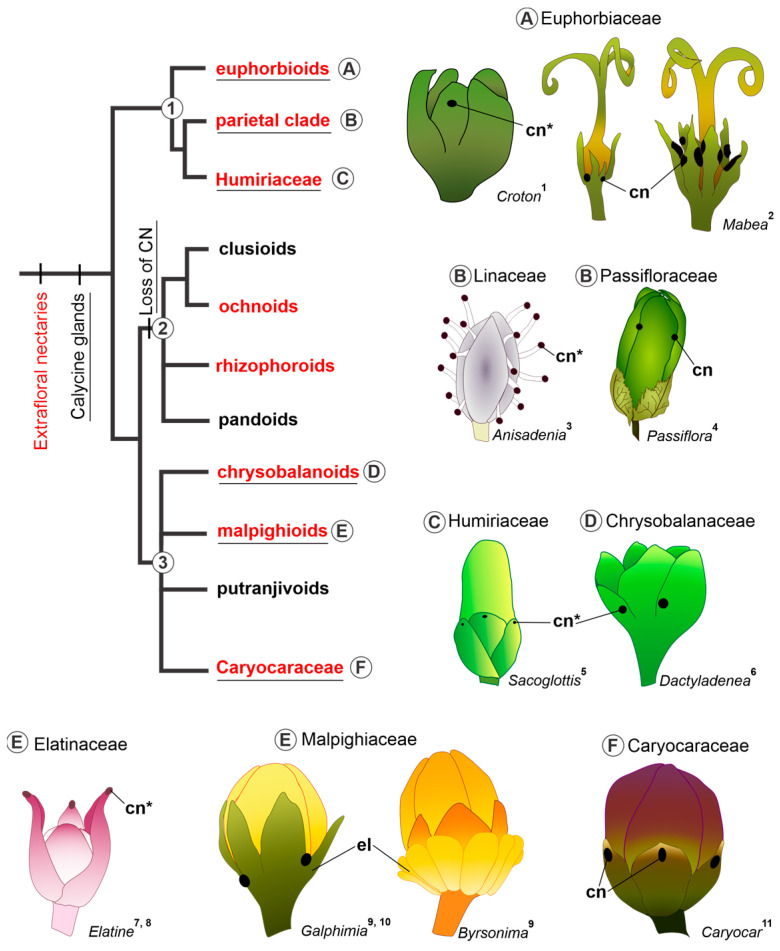
Distribution of extrafloral nectaries (written in red) and calycine nectaries in the families of Malpighiales (underlined), presented in a topology based on Xi et al. [8]. Capital letters link each subclade to the respective illustration. For extrafloral nectaries, only those arranged in vegetative organs were considered, according to Weber et al. [5]. Note that calycine nectaries are not described for clade 2. Superscript numbers after the genera whose flowers are illustrated indicate the data source: ^1^ Farias et al. [30]; ^2^ Almeida [29]; ^3^ Narayana and Rao [31]; ^4^ Knapp and Mallet [32]; ^5^ Wurdack and Zartman [36]; ^6^ Prance and White [37]; ^7^ Niedenzu [42]; ^8^ Bonifácio et al. [39]; ^9^ Castro et al. [24]; ^10^ This work; ^11^ Oliveira [40]. Abbreviations: cn, calycine nectary; cn*, calycine nectary not confirmed by histochemical tests; el, elaiophore. We applied fantasy colours.

In Figure 5, we compile data about the occurrence of EFNs and calycine glands in the Malpighiales tree. We also illustrate the position and distribution of calycine glands (whether nectaries or elaiophores) in different genera of clade 1 (euphorbioids, parietal clade, and Humiriaceae) and clade 3 (chysobalanoids, malpighioids, putranjivoids, and Caryocaraceae); clade 2 is not illustrated because of the loss of calycinal glands.

## 3. Discussion

### 3.1. Ontogenetic Origin of Elaiophores in Malpighiaceae 

The vasculature and ontogeny of elaiophores within basal clades of Malpighiaceae provide evidence for their calycine origin. Across all species examined in this study and those documented in the literature, elaiophores are consistently vascularised by lateral sepal traces [43,44,45]. These traces, in turn, originate directly from median sepal traces or derive from vascular complexes involving the calyx and corolla (in species that produce sepal-petal complexes) or even the androecium (in species with sepal–petal–antipetalous stamen complexes). Vascular sharing between two or more whorls and between adjacent sepals, including their respective glands, is a well-known pattern in the family [43,44,45]. The origin of vascular traces for elaiophores from the median sepal trace is first reported in this work and was only observed in *Lophanthera longifolia*.

According to the most recent phylogeny, which includes the two species analysed here, *Lophanthera* emerges as one of the earliest diverging genera within Malpighiaceae [18,19,20], with preliminary data from next-generation sequencing suggesting it to be the most basal genus in the family (Davis and Amorim, pers. comm.). Thus, the condition of *L. longifolia* may be plesiomorphic, given its simplicity compared to other species. This interpretation aligns with the glandular origin on the margins of the sepals, as evidenced by the ontogenetic analysis of elaiophores conducted in this study, signifying an autapomorphy specific to this species.

Conversely, the other genera of the basal clades acmantheroid [45], byrsonimoid, and galphimioid (except for *L. longifolia*) exhibit sharing of lateral sepal traces between adjacent sepals, which may also indicate a plesiomorphic condition. The morphological description of basally fused elaiophores in these Malpighiaceae lineages [17] also reinforces that there is a conation of the sepals. Furthermore, the initial stages of elaiophore ontogeny and vascular sharing between adjacent sepals align with the vestigial existence of a gamosepalous calyx [45]. The occurrence of a gamosepalous calyx connects Malpighiaceae to other families of Malpighiales that also have connate sepals, such as Caryocaraceae [4,46], Elatinaceae [39], Euphorbiaceae [47], and Rhizophoraceae [4].

The presence of vascular complexes in the procambial stage during floral development and their occurrence in more derived clades [43,44] reflect the floral conservatism of Malpighiaceae. As Anderson [13] highlighted regarding the overall flower morphology, conservatism is evident not only externally but also internally in the floral vasculature. It also indicates that floral morphology, including the elaiophores, may not be as labile as suggested in the literature [16,18,25]. Such insights reinforce the traditional interpretation that vascular structures represent the most conserved aspect of the flower [27,28], suggesting that the vascular system is the last to undergo evolutionary modification [48].

The vasculature pattern of the calyx provides insights into the suppression of elaiophores in the Malpighiaceae, particularly discernible through the distribution of lateral sepal traces. The presence of elaiophores is associated with basipetal traces originating from the vascular complexes and traces that irrigate the sepals. The relationship between elaiophores and basipetal traces is observed in species in which the glands exhibit vasculature in their proximal region, below the point of emission of vascular traces, a fact evident in *Galphimia australis*, *Byrsonima stipulacea* (both in this work), and *Pterandra pyroidea* [45]; in these species, the presence of basipetal traces between adjacent sepals suggests vascular migration to the glands, occurring in both glandular and eglandular morphs. Conversely, in *G. brasiliensis*, basipetal traces are notably absent, likely indicating a more extended evolutionary history of gland loss accompanied by the reduction in vascular traces. This vascular analysis aligns with the hypothesis proposed by Castro et al. [24], suggesting that gland suppression in *G. brasiliensis* resulted from loss rather than fusion. The reduction of elaiophores appears to be the event preceding their suppression in some lineages and allows the prediction of their disappearance in specific lineages, including some species of *Byrsonima*. 

From another point of view, in *Coleostachys genipifolia* (a monospecific genus with eglandular sepals), vascular traces occur in the margin of the receptacle basally to the emission of traces [45], suggesting a relatively recent evolutionary loss. Thus, the relic of the vasculature of the glands can be interpreted as a phylogenetic signal. This type of phylogenetic signal is also observed in groups related to Malpighiaceae, such as *Caryocar* (see Figure 30 and Figure 43 of Dickison [46]). These traces may also have had their direction modified, expressing themselves acropetally according to the extension of the gland, like in *Janusia*, *Mascagnia*, and *Tetrapterys*, as described by Souto and Oliveira [43]. Thus, considering the existence of a relationship between basipetal traces and elaiophores, there is further evidence that elaiophores constitute phylogenetically well-established and very well-conserved structures in the floral evolution of Malpighiaceae.

### 3.2. Evolutionary Origin of Elaiophores in Malpighiaceae

**Figure 6:**
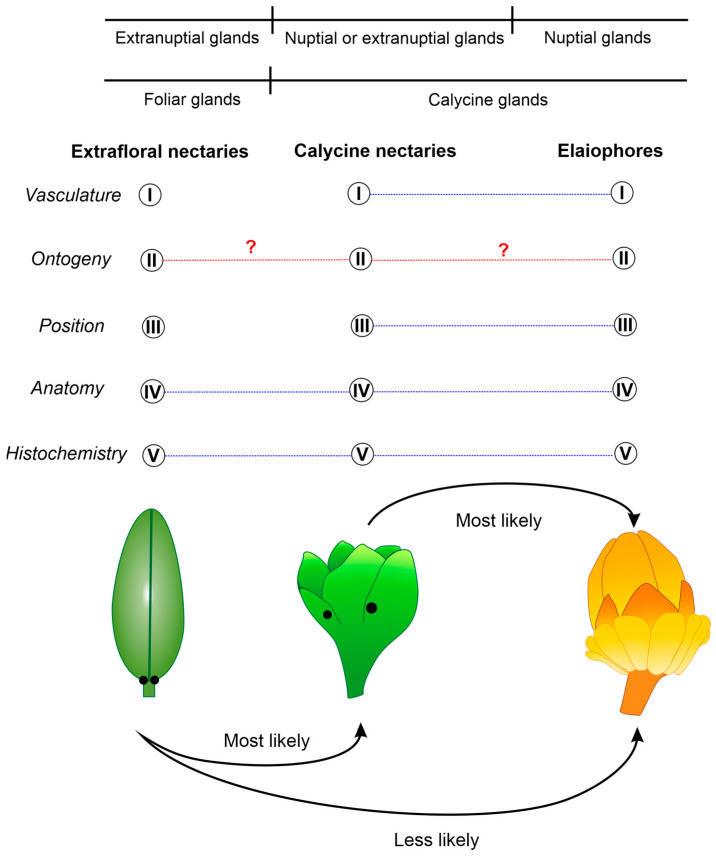
Similarity relationships between extrafloral nectaries, calycine nectaries, and elaiophores in Malpighiales adopting the homology criteria: (I) vasculature, (II) ontogeny, (III) position, (IV) anatomy, and (V) histochemistry. The dashed lines between the numbers of each analysed criterion indicate similarity (the question mark indicates insufficient data). The continuous lines show that calycine nectaries and elaiophores are subtypes of calycine glands mostly performing a nuptial function; the extranuptial function is typical of extrafloral nectaries (e.g., in leaves of Malpighiales), but can also be performed by calycine nectaries in certain species. In the lower part of the figure, possible evolutionary relationships between extrafloral nectaries, calycine nectaries, and elaiophores are shown.

To explore the evolutionary origins of elaiophores in Malpighiaceae, it is imperative to apply homology criteria encompassing vasculature, ontogeny, position, anatomy, and histochemistry across the structures under scrutiny—extrafloral nectaries (NEFs, exclusively present in vegetative organs), calycine nectaries (occurring on sepals), and elaiophores (see Figure 6). The analysis of original data from this study, alongside insights gleaned from the literature on vasculature and floral ontogeny across various species of Malpighiaceae and other families of Malpighiales, reveals striking similarities among these three gland types. 

The presence of vascular tissue irrigating the extrafloral nectaries (EFNs) and elaiophores is well documented in Malpighiaceae, establishing a connection between these two gland types. Nectaries, regardless of their ontogenetic origin, typically receive vasculature from both the xylem and phloem, a characteristic observed in elaiophores studied in representatives of this family [49,50]. However, the literature usually fails to address the origin of the vascular system of these structures, that is, from which organs their vasculature arises. EFNs originating from vegetative organs receive vascular traces from their respective organs of origin; for instance, those arising from leaves receive leaf traces. Similarly, floral nectaries are irrigated by vascular traces originating from various floral whorls, akin to the vasculature pattern observed in elaiophores of Malpighiaceae, which are vascularised by sepal traces, as evidenced in *Byrsonima* and *Galphimia* in this work. This scenario is likely analogous to the vasculature pattern observed in nectaries on sepals of other families of Malpighiales, such as Caryocaraceae [4] and Euphorbiaceae [29]. Consequently, nectaries on sepals and elaiophores in Malpighiaceae share a common origin: the sepals.

Although vasculature information is lacking for most calycine nectaries, the position serves as another criterion to be utilised. It reinforces the homology between calycine nectaries and elaiophores, as both constitute calycine glands—glands originating from the sepals.

Considering the ontogeny of elaiophores in the two basal lineages examined here and the presence of nectaries on leaves of *Banisteriopsis muricata* [51], both originate from cell divisions in the protodermis and ground meristem, and both have procambial strands. However, to date, we do not have ontogenetic information about nectaries on sepals in Malpighiales lineages which could clarify whether these glands share similar development. Nevertheless, it is plausible that all nectaries with a parenchymatous structure, whether present in vegetative organs (extrafloral) or associated with flowers (floral), follow the same ontogenetic progression, regardless of the family in which they occur. The homology between nectaries occurring in vegetative organs (EFNs) and those located in flowers (FNs) remains an unanswered question since the genetic bases that regulate their expression still need to be better understood [52].

The structure of nectaries in vegetative organs and calycine nectaries exhibits remarkable similarity across taxonomic groups: they consist of secretory parenchyma and vascular tissues surrounded by a uniseriate secretory epithelium, encapsulated by a cuticle [4,29,49]; we found that all these traits are similar for elaiophores in Malpighiaceae. Furthermore, foliar nectaries and elaiophores of *Diplopterys pubipetala* share the same cell machinery related to synthesis processes, with mitochondria-rich cytoplasm, rough and smooth endoplasmic reticulum, and plastids [11,50].

Regarding gland exudates, even scarce histochemical data reveal the presence of sugars in elaiophores and lipids in nectaries, although oils and sugars are predominant, respectively. The presence of lipids in foliar nectaries of Malpighiaceae is reported for different genera, such as *Galphimia* [24], *Diplopterys* [11], and *Banisteriopsis* [49]. In elaiophores, there are reports of mixed exudates containing lipids, proteins, and polysaccharides [11,12,49,50], or lipids and sugar [24]. In the Malpighiaceae genera distributed in the Old World, where oil-collecting bees are absent, calycine glands function as nectaries without any lipid content [25]. It is significant, therefore, that these glandular types possess the synthetic machinery for both oils and nectar. Consequently, from an anatomical or secretion biology perspective, there is no indication that elaiophores cannot be preceded by extranuptial nectaries, as we hypothesise in Malpighiaceae.

It is noteworthy that ecological data regarding the relationships between Malpighiaceae species and ants suggest an increase in fruiting rates facilitated by the patrolling of these insects in floral buds of *Byrsonima crassifolia* [53], a species of the byrsonimoid clade characterised by the absence of extrafloral nectaries (similar to galphimioid species) [17]. In *B. crassifolia*, Sazima and Sazima [54] and Sigrist and Sazima [55] noted that while elaiophores typically function to attract pollinators, as commonly observed, these glands can also secrete substances that attract ants [53], such as glucose, as observed by Possobom and Machado [50] in *Byrsonima coccolobifolia* and *Banisteriopsis variabilis*. Indeed, depending on the phenological period of the plant, there is a shift in the concentrations of exudates, with sugars predominating when they serve a defensive function similar to nectaries in vegetative organs and oils predominating when they serve a nuptial function in attracting pollinators. Histochemical analyses conducted across phenological phases of these plants may provide further insight and corroborate the hypothesis regarding the origin of elaiophores from sepal nectaries.

Data on secretion biology, vasculature, and floral development are scarce for the families and species in which calycine nectaries are described in Malpighiales (see Table 1). In Linaceae, the calycine glands are vascularised from lateral sepal traces [31], which appears to be the case in *Mabea* (Euphorbiaceae) [29]. Even for *Caryocar* (Caryocaraceae), a genus in which there are data on floral vasculature, there is no record of the origin of the traces that vascularise the glands [4]. However, the calycine glands in *Caryocar* are sepal nectaries since the presence of nectar is reported by studies that deal with ecological relationships, such as patrolling by ants [41,56].

When considering the occurrence of nectaries in vegetative organs (EFNs) and calycine nectaries within a phylogenetic framework of Malpighiales, it becomes apparent that, for parsimony, the most proximal relationship likely exists between elaiophores and sepal nectaries. The presence of calycine glands on the abaxial surface of the sepals is reported for the following families of Malpighiales: Euphorbiaceae [29,30,57], Linaceae [31], Passifloraceae [32,33,34], Humiriaceae [35,36], Chrysobalanaceae [37,38], Caryocaraceae [4,40,41,56], and Elatinaceae [39,42]. Additionally, evidence of secretory structures was also observed on the margins of the sepals of *Bergia perennis* and the apex of the sepals of *Elatine gratioloides* [39]. Consequently, the occurrence of calycine glands (without specifying the type of secretion) encompasses two of the three main lineages of Malpighiales, clades 1 and 3 of Xi et al. [8].

The phylogenetic distribution of nectaries in vegetative organs (extrafloral) among Malpighiales lineages is observed across all three clades of the order [5,8,52] (see Figure 5). The presence of EFNs in different families of Malpighiales is well known [5,39,52,58,59] and comprises approximately a quarter of all species with EFNs described among angiosperms [52]. Hence, the presence of calycine nectaries and EFNs can be considered plesiomorphic for Malpighiales.

According to Vogel [15], the widespread occurrence of EFNs in Malpighiaceae indicates that they are the plesiomorphic structure from which elaiophores evolved (see Vogel [15], p. 137). Therefore, it is common to consider that elaiophores and foliar nectaries (EFNs) are homologous [14,24,25,49,60]. This proposition is supported by studies focusing on these secretory structures in Malpighiaceae. For Paleotropical lineages, the exclusive presence of carbohydrates in the secretion is known, such as *Hiptage benghalensis* (L.) Kurz [61] and *Acridocarpus* [25], constituting calycine nectaries. According to Guesdon et al. [25], the presence of carbohydrates in the exudate of elaiophores indicates a relationship between them and extrafloral nectaries, also supporting the homology hypothesis. 

However, if both extrafloral nectaries and calycine nectaries are considered plesiomorphic across all lineages of Malpighiales, then the transition of nectaries from vegetative organs to the flower likely took place in the common ancestor of all lineages within the order. Consequently, calycine nectaries serve as the precursors to elaiophores.

Notably, from a phylogenetic perspective, not all relationships between clades within Malpighiales are thoroughly resolved, a phenomenon often attributed to the rapid origin and diversification of the group during the Cretaceous [8,62]. During this same period, ants were already foraging in trees [63], and it is known that extrafloral nectaries can indirectly increase the diversification rate in the clades in which they occur [64]. Consequently, investigations focusing on the evolutionary dynamics of floral nectaries with extranuptial functions, such as calycine nectaries, hold promise for advancing our understanding of Malpighiales. However, there is a lack of studies comprehensively examining the distribution, origin, and secretory characteristics of calycine glands across Malpighiales. 

## 4. Materials and Methods

### 4.1. Sampling 

We selected three individuals for each genus from the galphimioid and byrsonimoid clades (Table 2). We selected these two clades because they are basal in Malpighiaceae, and their intergeneric relationships are well supported [18]. Under these conditions, plesiomorphic states for the family are expected to be sampled. In the case of genera or species that exhibit glandular and eglandular morphs (flowers with and without elaiophores, respectively), we sampled both forms and included species of the same genus that do not present this variation. For the analysis of floral vasculature, the material consisted of floral buds close to anthesis from herbal material or field collections. To determine the origin of elaiophores, in addition to the vasculature, we considered the ontogeny of these structures. For this, we selected *Verrucularia glaucophylla*, *Lophanthera lactescens* (galphimioid clade), and *Byrsonima triopterifolia* (byrsonimoid clade).

### 4.2. Anatomical Analysis

The material from exsiccates was subjected to the drying reversal process, dehydrated in an ethyl series, and kept in 70% ethanol (Smith and Smith [65], modified by Mello et al. [66]). The material collected in the field was fixed in 37% formaldehyde, acetic acid, and 50% ethanol (1:1:18 *v*/*v*) for 48 h [67] and stored in 50% ethanol. Afterwards, all samples were dehydrated in an ethanol series, embedded in historesin (Leica (2-hydroxyethyl)-methacrylate, Leica, Wetzlar, Germany) [68], and sectioned on a rotary microtome at 5–10 µm thickness. Sections were stained with 0.05% toluidine blue at pH 4.7 in acetate buffer [69], modified) and mounted in Entellan. The images were obtained using an Olympus CX41 microscope (Olympus Life Sciences, Tokyo, Japan) with an LC20 camera attached. They were then gathered and aligned using the Image Composite Editor 2015 software, edited in Corel Photo-Paint 2020, and organised in CorelDraw Graphics Suite 2020.

### 4.3. Terminological Framework

In this study, we adopted the topological terminology for nectaries proposed by Caspary [21], which classifies extrafloral nectaries (EFNs) as those situated outside the flower, typically in vegetative organs, and floral nectaries (FNs) as those found in flowers. Throughout our discussions, to underscore the positional criterion, we meticulously specified the location of the nectaries, indicating the organ in which they are situated. For instance, “foliar nectary” denotes a nectary specifically located in the leaf. Additionally, when addressing the functional aspect of nectaries, irrespective of their location, we utilised the terms “extranuptial” to denote a nectary with a protective function and “nuptial” to signify a nectary involved in attracting and rewarding pollinators, as proposed originally by Delpino [22].

Therefore, we adopted calycine glands to encompass elaiophores and nectaries on sepals. Hence, to maintain clarity and consistency, we employed the term elaiophore to specifically denote the calycine glands of Malpighiaceae responsible for synthesising and releasing oil. Even in instances where the chemical composition of the secretion was unspecified, we designated the glandular structures occurring in the calyces of Neotropical species of the family as elaiophores.

The descriptions of vasculature followed the terminology adopted by Puri [48], Souto and Oliveira [43], Mello [44], and Bonifácio et al. [45]. By these references, the following terms are employed: (a) vascular trace, which refers to the vascular unit emitted to an organ that has not yet become individualised at the given height (e.g., a sepal trace is seen at the receptacle level); (b) vascular complex, which is used when the vascular unit will irrigate pieces form more than one floral whorl (e.g., a sepal–petal–antipetalous stamen complex is emitted by the stele and, in a higher level, divides and emits traces to a sepal, a petal, and a stamen opposite that petal); and (c) basipetal traces, which designate those vascular units emitted in a higher position in the flower that have a basipetal orientation, characterised by xylem facing the external surface of the organ. 

Given that the primary aim of this study was to elucidate the evolutionary origins of Malpighiaceae elaiophores, our analyses of floral vasculature were limited to the calyx, a whorl directly associated with these glands. However, when the calyx and corolla shared vasculature, their vascular traces were described. Similarly, when the calyx, corolla, and androecium were presented, they all had some relation with the calycine glands. In all cases, we did not analyse the gynoecium vasculature. 

### 4.4. Criteria for Homology Assessment

In our investigation of the homology relationships between nectaries and elaiophores, we employed the following criteria: (I) vasculature (the presence of vascular bundles and their origin); (II) ontogeny; (III) position (based on topological correspondence); (IV) anatomy; and (V) histochemistry. The first two criteria were explicitly addressed in this study concerning basal clades of Malpighiaceae, filling a gap in the literature about the family. The other three criteria were evaluated from findings in the literature on Malpighiaceae and closely related families, following the most recent phylogeny of Malpighiales [8]. We analysed taxonomic descriptions for gland position data and anatomical studies for collecting data about structure and histochemistry. 

## 5. Conclusions

Applying diverse homology criteria, encompassing the vasculature and ontogeny of elaiophores in galphimioid and byrsonimoid taxa (Malpighiaceae), in comparison with findings in the literature on calycine and extrafloral nectaries across various lineages of Malpighiales, led us to infer that the presence of EFNs and calycine nectaries represents a plesiomorphic trait for the order. We also suggest that the transition from NEFs to floral nectaries (NFs) likely occurred in ancestral lineages of Malpighiales, predating the emergence of Malpighiaceae. Consequently, the elaiophores in Malpighiaceae likely evolved from calycine nectaries, indicating an evolutionary history considerably older than previously stated in the literature.

## Figures and Tables

**Table 1:** Occurrence of nectary-like glands in the sepals of Malpighiales. (present: +; unavailable data: ?).

Family	Species	Occurrence of Nectary (Evidence)	Distribution of Glands in Sepals	Position in the Sepal	Number of Glands per Sepal	Ontogenetic Origin of the Gland	Trace that Vascularises the Gland	Reference
Euphorbiaceae	*Mabea* *fistulifera*	+ (histochemistry)	All sepals	Margin (between two sepals)	1 to several	?	Lateral trace of sepal *	Almeida [29]
	*Croton rizzinii*	?	Some of the sepals	Median region	1		?	Farias et al. [30]
Linaceae	*Acioa edulis*	?	3 of 5 sepals (inner sepals do not have)	Margin	Several	?	Lateral trace of sepal	Narayana and Rao [31]
Passifloraceae	*Passiflora* *macdougaliana*	+ (ants visiting)	3 or all	All the margin	1 or 2	?	?	Knapp and Mallet [32]
	*Passiflora edulis*				2 or 3			Bernacci et al. [33]
					1 or 2			Shivanna [34]
Humiriaceae	*Sacoglottis* *amazonica*	?	?	Margin or apex	Several	?	?	Cuatrecasas [35] Wurdack and Zartman [36]
Chrysobalanaceae	*Dactyladenia* *Acioa*	?	?	Margin or median region	1 to several	?	?	Prance and White [37]
	*Hirtella*	?	?	Margin	Several		?	Asprino and Amorim [38]
Elatinaceae	*Bergia perennis*	?	All	Margin	1 or 2	?	Median trace of sepal	Bonifácio et al. [39]
	*Elatine* *gratioloides*			Apex	1		Median trace of sepal	
Caryocaraceae	*Caryocar* *brasiliense*	+ (ants visiting)	?	Median region	1	?	?	Oliveira [40] Oliveira and Freitas [41] Machado et al. [2] Matthews and Endress [4]

* The author does not mention sepal vasculature, but the analysis of some figures allows us to infer this condition.

**Table 2:** Samples of the clades galphimioid and byrsonimoid used in this work (calyx status: EG eglandular calyx, GL glandular calyx).

Cla-de	Species	Voucher	Samples	Provenance	Calyx Status
byrsonimoid	*Byrsonima stipulacea* A.Juss.	Amorim et al. 3355 (CEPEC)	dried	Espírito Santo, Brazil	EG
		D. Sucre 8353 (CEPEC)	dried	Espírito Santo, Brazil	EG
		Jardim et al. 4327 (CEPEC)	dried	Bahia, Brazil	EG
		V. Demuner et al. 3622 (CEPEC)	dried	Espírito Santo, Brazil	GL
		Folli, D.A. s.n. (CEPEC)	dried	Espírito Santo, Brazil	GL
		Kollmann et al. 2536 (CEPEC)	dried	Espírito Santo, Brazil	GL
	*Byrsonima triopterifolia* A.Juss.	Amorim 10797 (CEPEC)	fixed	Bahia, Brazil	GL
		Amorim 10798 (CEPEC)	fixed	Bahia, Brazil	GL
		Amorim 10799 (CEPEC)	fixed	Bahia, Brazil	GL
	*Blepharandra hypoleuca* Griseb.	Martinelli 17280 (CEPEC)	dried	Amazonas, Brazil	GL
		Forzza 6559 (CEPEC)	dried	Amazonas, Brazil	GL
	*Diacidia aracaensis* W.R.Anderson	Forzza 6561 (CEPEC)	dried	Amazonas, Brazil	GL
		Amorim 8617 (CEPEC)	dried	Amazonas, Brazil	GL
galphimioid	*Galphimia australis* Chodat	Vasco 20495 (CEPEC)	dried	Minas Gerais, Brazil	EG
		Queiroz 12643 (CEPEC)	dried	Rio Grande do Sul, Brazil	EG
		Poliquesi 495 (CEPEC)	dried	Santa Catarina, Brazil	EG
		Irwin 26193 (CEPEC)	dried	Minas Gerais, Brazil	GL
		Rezende 2182 (HUEFS)	dried	Minas Gerais, Brazil	GL
	*Galphimia brasiliensis* A.Juss.	Cardoso 916 (CEPEC)	dried	Bahia, Brazil	EG
		Santos 3070 (CEPEC)	dried	Bahia, Brazil	EG
		Bastos 162 (CEPEC)	dried	Bahia, Brazil	EG
	*Lophanthera lactescens* Ducke	Bonifácio 32 (BHCB)	fixed	Minas Gerais, Brazil	GL
	*Lophanthera longifolia* (Kunth) Griseb.	Fraga 3014 (CEPEC)	dried	Pará, Brazil	GL
		Santos 23 (CEPEC)	dried	Amazonas, Brazil	GL
	*Spachea elegans* A.Juss.	Martinelli 17422 (CEPEC)	dried	Roraima, Brazil	GL
	*Verrucularia glaucophylla* Juss.	Amorim et al. 10944 (CEPEC)	fixed	Bahia, Brazil	GL
		Amorim et al. 10947 (CEPEC)	fixed	Bahia, Brazil	GL
		Amorim et al. 10948 (CEPEC)	fixed	Bahia, Brazil	GL

## Data Availability

The original contributions presented in the study are included in the article and Supplementary Material; further inquiries can be directed to the corresponding author.

## Supplementary Materials

The following supporting information can be downloaded at: https://www.mdpi.com/article/10.3390/plants13121654/s1, Figure S1: Calycine vasculature of *Galphimia australis*, *G. brasiliensis*, and *Verrucularia glaucophylla* (galphimioid clade) with sepal–petal complexes; Figure S2: Calycine vasculature of *Byrsonima stipulacea* (byrsonimoid clade) with sepal–petal complexes; Figure S3: Calycine vasculature of *Blepharandra hypoleuca* (byrsonimoid clade) with sepal–petal complexes; Figure S4: Calycine vasculature of *Spachea elegans* and *Lophanthera lactescens* (galphimioid clade) with sepal–petal–antipetalous stamen complexes; Figure S5: Calycine vasculature of *Lophanthera longifolia* (galphimioid clade) with median sepal traces and one sepal–petal–antipetalous stamen complex; Figure S6: Calycine vasculature of *Diacidia aracaensis* (byrsonimoid clade) with median sepal–petal complexes.
