# Supplementary material for: Unravelling the Homology between Calycine Glands in Malpighiales: New Data from Basal Malpighiaceae
Source: Plants (Basel). 2024 Jun 14;13(12):1654. doi: 10.3390/plants13121654 (PMC11207516; doi:10.3390/plants13121654)
Supplement: Supplementary file 1 [file plants-13-01654-s001.zip › plants-3008056-supplementary.pdf]

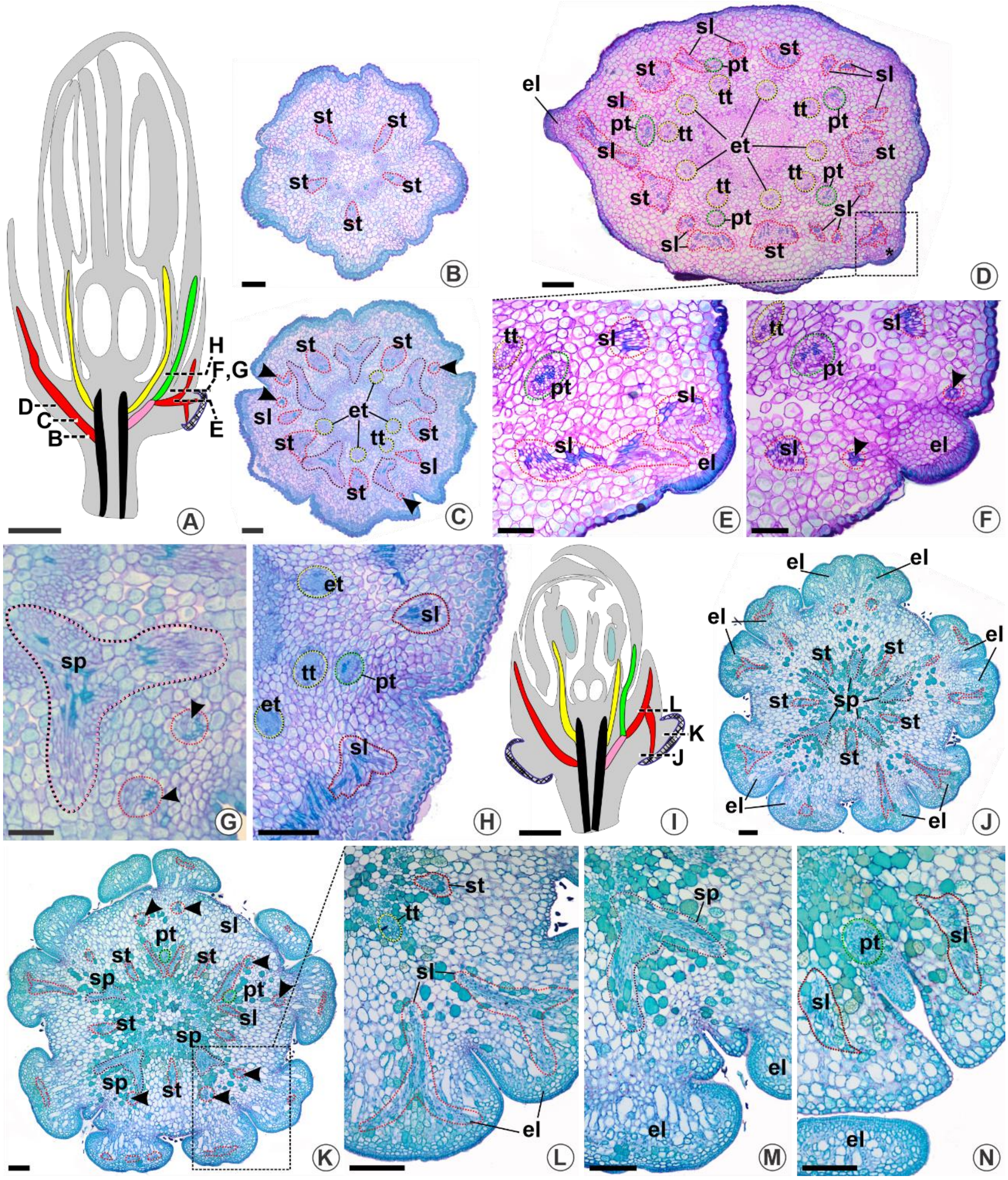

Figure S1. Calycine vasculature of *Galphimia australis* (**A**-**G**), *G*. *brasiliensis* (**H**) and *Verrucularia glaucophylla* (**I**-**N**). The hatched areas represent the epidermis of elaiophores, while colours represent vascular cylinder (black), sepal-petal complex (pink), sepal trace (red), petal trace (green), stamen trace (yellow). (**A**, **I**) Diagrams of median longitudinal reconstruction of the floral bud showing the vasculature of the elaiophores (black dashed lines point to the approximate position of the cross sections indicated by capital letters). (**B**-**H**, **J**-**N**) Photomicrographs of floral buds in cross section at different heights. (**B**-**H**) Emission of the median sepal traces and sepal-petal complexes; each sepal-petal complex branches originating the petal trace internally and, laterally, two lateral sepal traces, which irrigate the elaiophores; in **E**, note the lateral traces of adjacent sepals irrigating the same elaiophore; in **F**-**G**, note the presence of basipetal traces respectively in glandular and eglandular morphs of *G. australis* (arrowheads). (**J**-**N**) Emission of the median sepal traces and sepal-petal complexes; in **K**, note the presence of basipetal traces (arrowheads); in **L**, note that each lateral sepal trace irrigates the elaiophore of that specific sepal. Abbreviations: el, elaiophore; et, antisepal stamen trace; pt, petal trace; sl, lateral sepal trace; sp, sepal-petal complex; st, median sepal trace; tt, antipetalous stamen trace. Scale bars = 200µm.

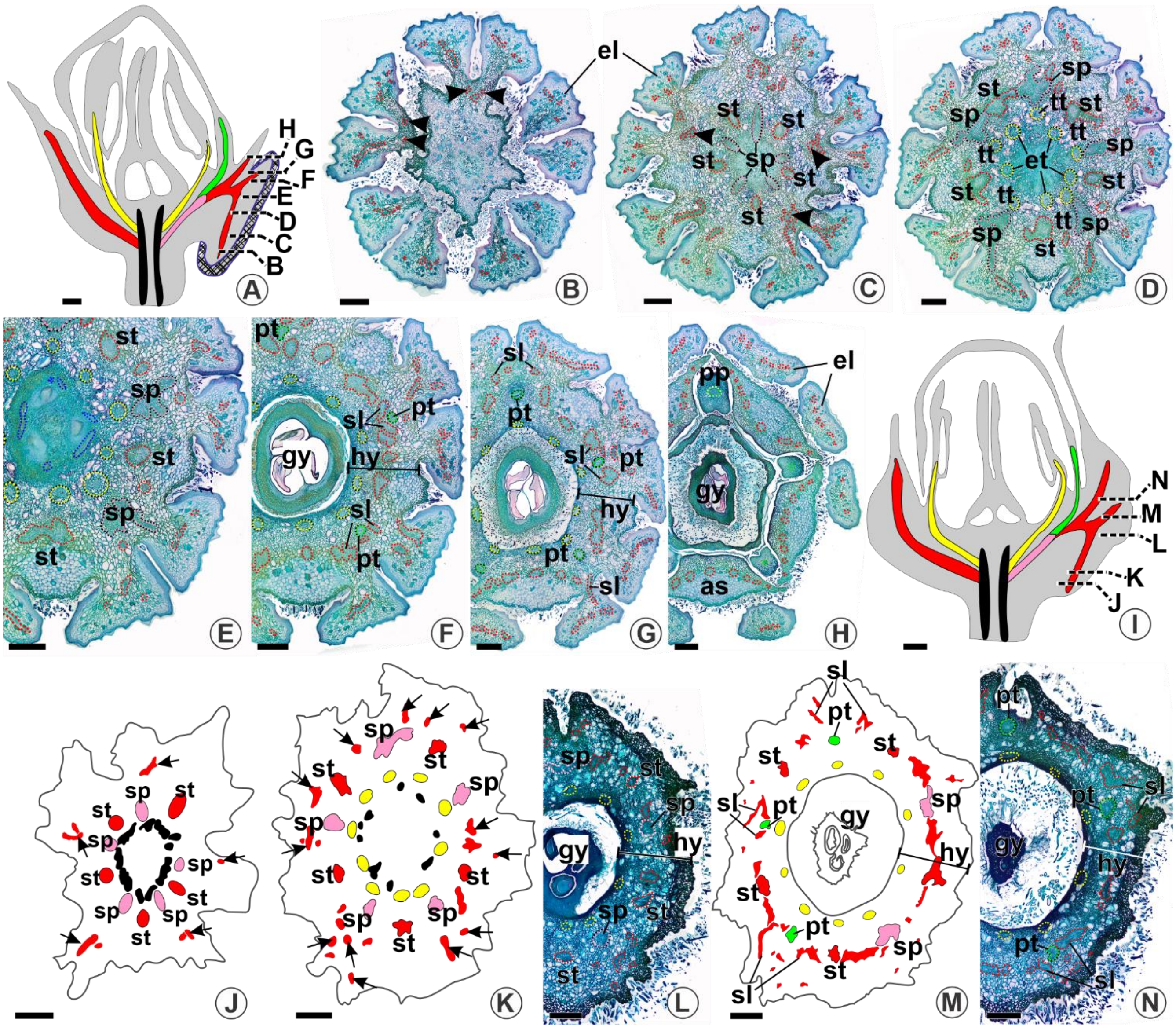

Figure S2. Calycine vasculature of glandular (**A**-**H**) and eglandular (**I**-**N**) morphs of *Byrsonima stipulacea*. The hatched area represents the epidermis of an elaiophore, while colours represent vascular cylinder (black), sepal-petal complex (pink), sepal trace (red), petal trace (green), stamen trace (yellow). (**A**, **I**) Diagrams of median longitudinal reconstruction of the floral bud showing the vasculature of the elaiophores (black dashed lines point to the approximate position of the cross sections indicated by capital letters). (**B**-**H**, **L**, **N**) Photomicrographs of floral buds in cross section at different heights. (**J**-**K**, **M**) Diagrams of cross sections highlighting the vasculature. (**B**-**H**) Emission of the median sepal traces and sepal-petal complexes (arrowheads: basipetal traces); each sepal-petal complex branches near the margin of the receptacle; from **E** upwards, note the lateral sepal traces irrigating the elaiophores; in **F**-**G**, note the occurrence of the hypanthium. (**J**-**N**) Emission of the median sepal traces and sepal-petal complexes in the eglandular morph; even in the absence of the elaiophores, note the vasculature in the margin of the receptacle (arrows); in **M**-**N**, note the occurrence of the hypanthium. Abbreviations: as, anterior sepal; el, elaiophore; et, antisepalous stamen trace; gy, gynoecium; hy, hypanthium; pp, posterior petal; pt, petal trace; sl, lateral sepal trace; sp, sepal-petal complex; st, median sepal trace; tt, antipetalous stamen trace. Scale bars = 400µm.

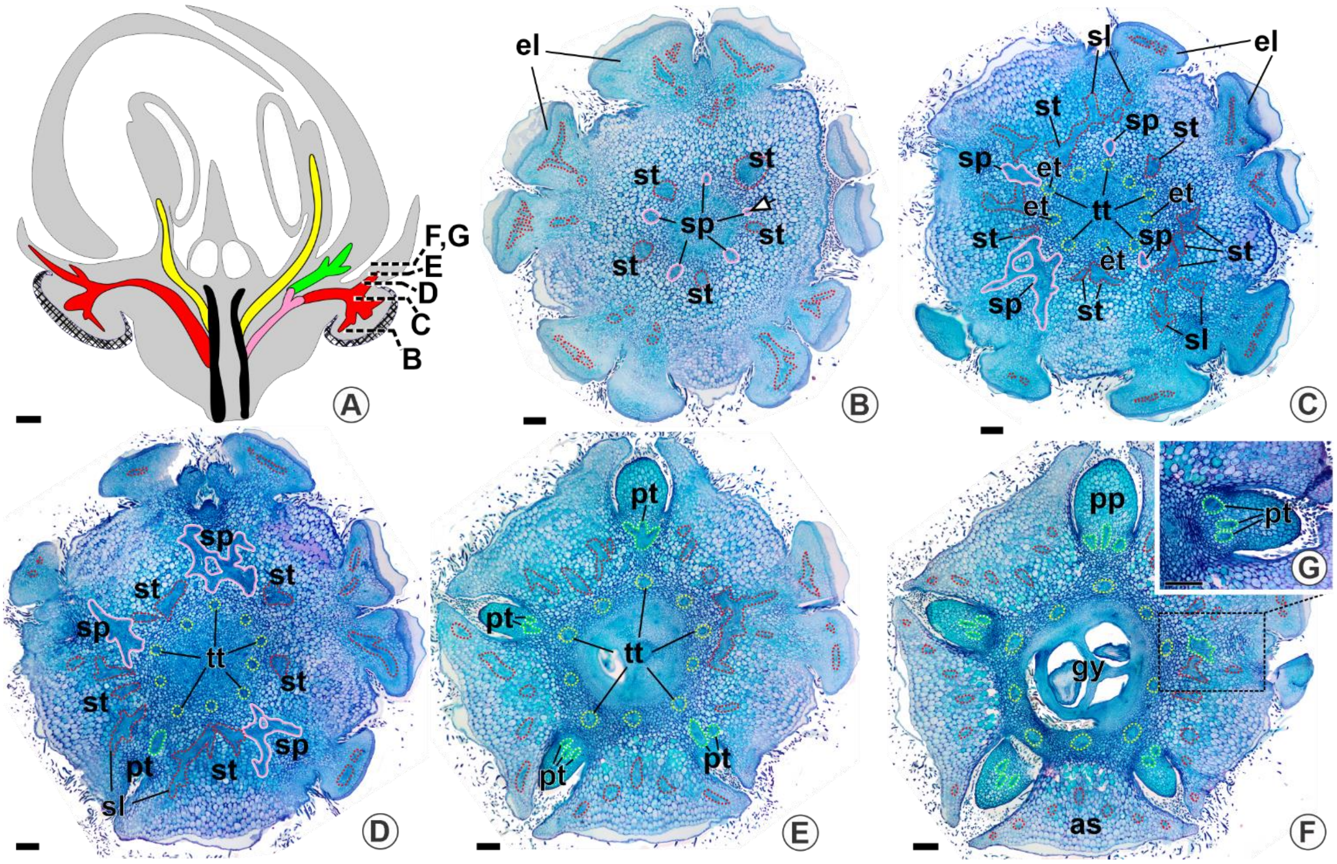

Figure S3. Calycine vasculature of *Blepharandra hypoleuca* (A-G). The hatched areas represent the epidermis of elaiophores, while colours represent vascular cylinder (black), sepal-petal complex (pink), sepal trace (red), petal trace (green), stamen trace (yellow). (**A**) Diagram of median longitudinal reconstruction of the floral bud showing the vasculature of the elaiophores (black dashed lines point to the approximate position of the cross sections indicated by capital letters). (**B**-**G**) Photomicrographs of floral buds in cross section at different heights. (**B**-**G**) Emission of the median sepal traces and sepal-petal complexes; from **C** upwards, note that lateral sepal traces originate from sepal-petal complexes and irrigate the elaiophores. Abbreviations: as, anterior sepal; el, elaiophore; et, antisepalous stamen trace; gy, gynoecium; pp, posterior petal; pt, petal trace; sl, lateral sepal trace; sp, sepal-petal complex; st, median sepal trace; tt, antipetalous stamen trace. Scale bars = 200µm.

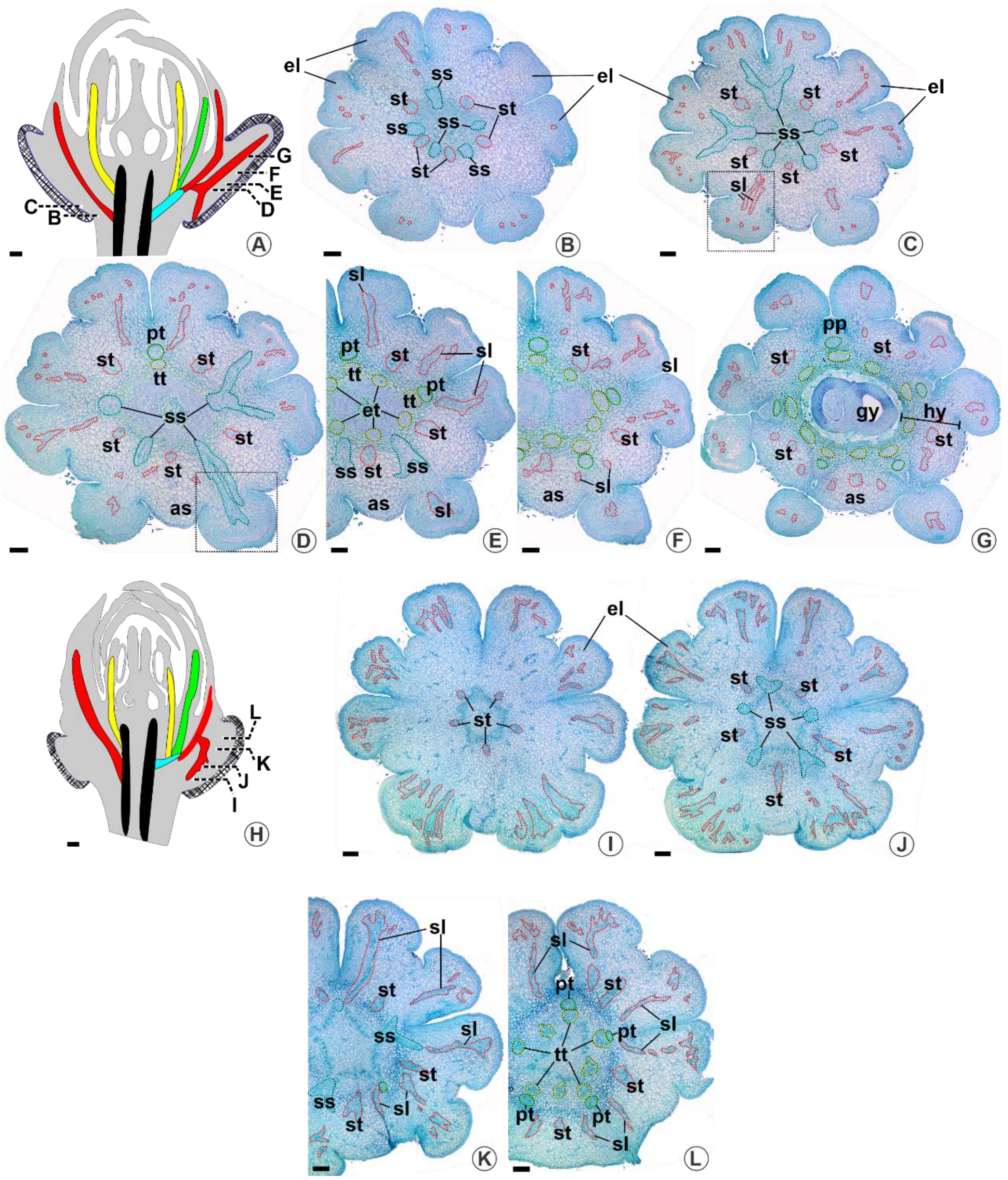

Figure S4. Calycine vasculature of *Spachea elegans* (**A**-**G**) e *Lophanthera lactescens* (**H**-**L**). The hatched areas represent the epidermis of elaiophores, while colours represent vascular cylinder (black), sepal-petal-antipetalous stamen complex (light blue), sepal trace (red), petal trace (green), stamen trace (yellow). (**A**, **H**) Diagrams of median longitudinal reconstruction of the floral bud showing the vasculature of the elaiophores (black dashed lines point to the approximate position of the cross sections indicated by capital letters). (**B**-**G**, **I**-**L**) Photomicrographs of floral buds in cross section at different heights. (**B**-**G**) Emission of the median sepal traces and sepal-petal-antipetalous stamen complexes; from **D** upwards, observe that after each complex branches towards the margin of the receptacle, an antipetal stamen trace is individualized from it internally, and a petal trace externally; laterally, two lateral sepal traces are emitted and irrigate two adjacent elaiophores; in **C** and **D**, note that the sepals adjacent to the anterior sepal receive four lateral sepal traces; in **G**, note the occurrence of the hypanthium. (**I**-**L**) Emission of the median sepal traces and sepal-petal-antipetalous stamen complexes; from **K** upwards, note lateral sepal traces irrigating the elaiophores. Abbreviations: as, anterior sepal; el, elaiophore; et, antisepalous stamen trace; gy, gynoecium; hy, hypanthium; pp, posterior petal; pt, petal trace; sl, lateral sepal trace; ss, sepal-petal-antipetalous stamen; st, median sepal trace; tt, antipetalous stamen trace. Scale bars = 200µm.

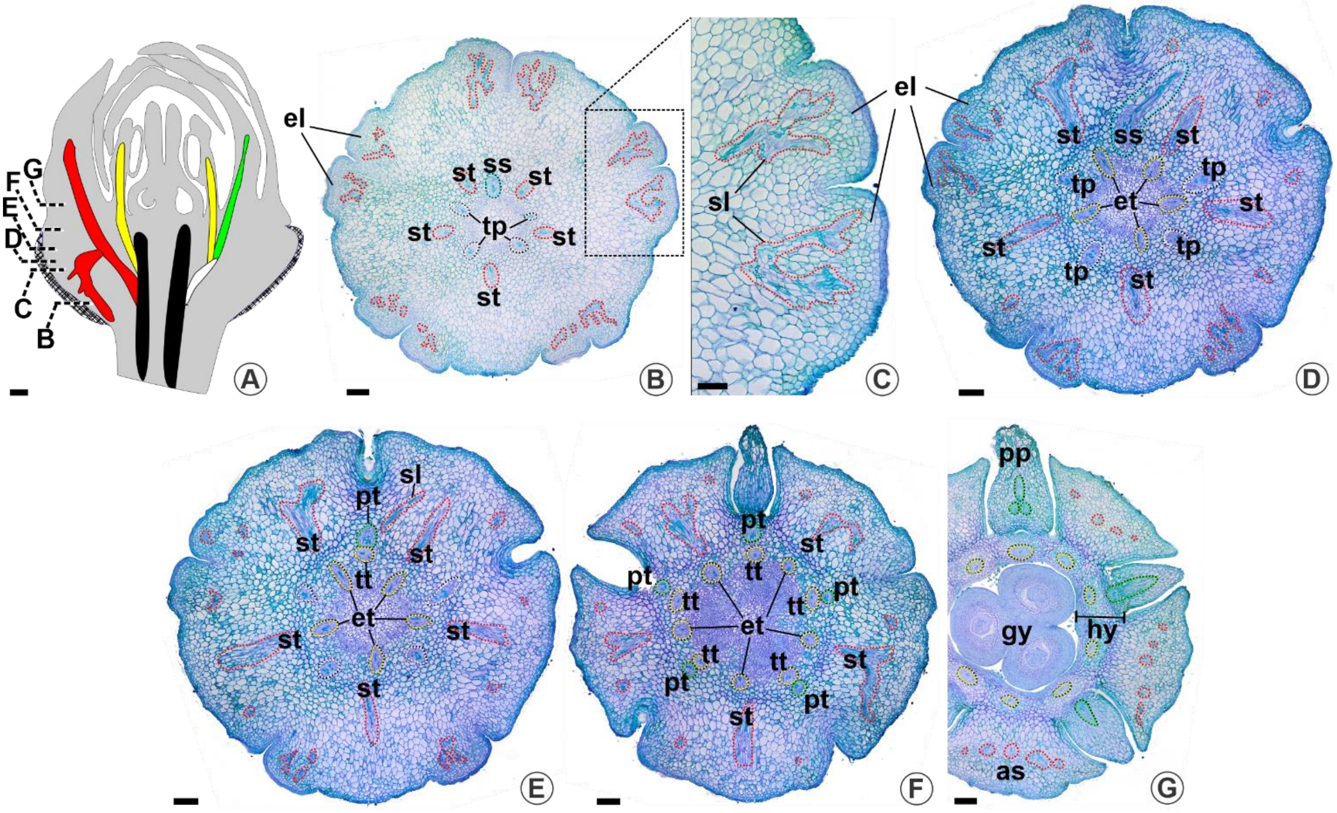

Figure S5. Calycine vasculature of *Lophanthera longifolia*. The hatched areas represent the epidermis of elaiophores, while colours represent vascular cylinder (black), sepal-petal-antipetalous stamen complex (white), sepal trace (red), petal trace (green), stamen trace (yellow). (**A**) Diagram of median longitudinal reconstruction of the floral bud showing the vasculature of the elaiophores (black dashed lines point to the approximate position of the cross sections indicated by capital letters) (**B-G**) Photomicrographs of floral buds in cross section at different heights. (**B-G**) Emission of the median sepal traces and sepal-petal-antipetalous stamen complexes; from **C** upwards, note the lateral sepal traces irrigating the elaiophores; in **G**, note the occurrence of the hypanthium. Abbreviations: as, anterior sepal; el, elaiophore; et, antisepalous stamen trace; gy, gynoecium; hy, hypanthium; pp, posterior petal; pt, petal trace; sl, lateral sepal trace; ss, sepal-petal-antipetalous stamen; st, median sepal trace; tp, sepal-petal-antipetalous stamen complex; tt, antipetalous stamen trace. Scale bars = 200µm.

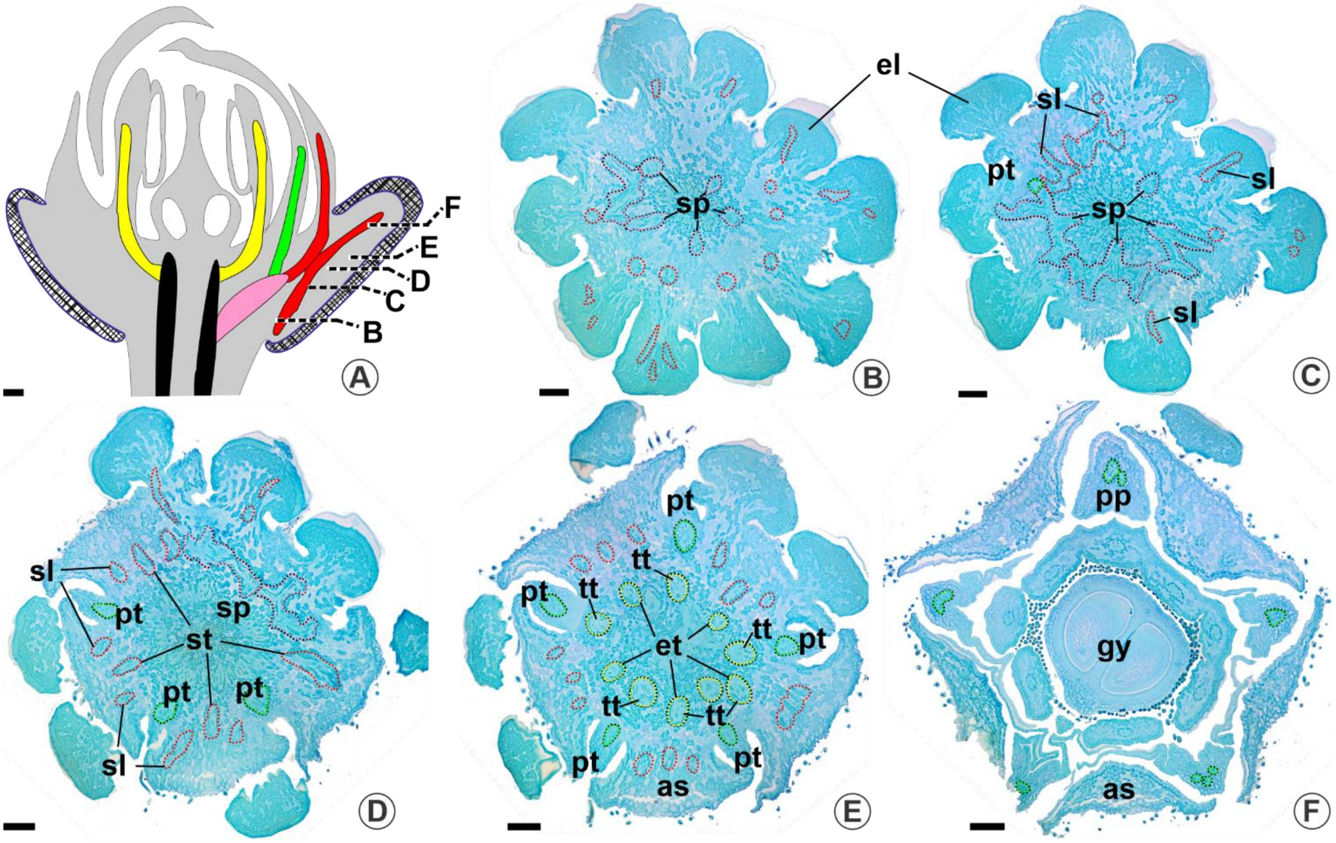

Figure S6. Calycine vasculature of *Diacidia aracaensis*. The hatched areas represent the epidermis of elaiophores, while colours represent vascular cylinder (black), sepal-petal complex (pink), sepal trace (red), petal trace (green), stamen trace (yellow). (**A**) Diagram of median longitudinal reconstruction of the floral bud showing the vasculature of the elaiophores (black dashed lines point to the approximate position of the cross sections indicated by capital letters). (**B**-**F**) Photomicrographs of floral buds in cross section at different heights. (**B**-**F**) Emission of the median sepal-petal complexes; from **B** upwards, note the lateral sepal traces irrigating the elaiophores; in this species, note that these complexes also originate the median sepal and petal traces (from **C** upwards). Abbreviations: as, anterior sepal; el, elaiophore; et, antisepalous stamen trace; gy, gynoecium; pp, posterior petal; pt, petal trace; sl, lateral sepal trace; sp, sepal-petal complex; st, median sepal trace; tt, antipetalous stamen trace. Scale bars = 200µm.
